# Abolish Psychiatry, Abolish Neurology: Toward a Unified Discipline of Brain Medicine

**DOI:** 10.7759/cureus.84817

**Published:** 2025-05-26

**Authors:** Shaheen E Lakhan

**Affiliations:** 1 Medical Office, Click Therapeutics, Inc., New York, USA; 2 Department of Neurology, Western University of Health Sciences, Pomona, USA; 3 Department of Neurology, A.T. Still University School of Osteopathic Medicine in Arizona, Mesa, USA; 4 Department of Neurology, Morehouse School of Medicine, Atlanta, USA; 5 Department of Bioscience, Global Neuroscience Initiative Foundation, Miami, USA

**Keywords:** brain medicine, computational psychiatry, digital therapeutics, fda regulation, medical education, network neuroscience, neurology, psychiatry, reimbursement, transdiagnostic care

## Abstract

The longstanding division between psychiatry and neurology is a historical artifact, not a scientific necessity. Despite addressing the same organ, these fields operate as separate disciplines, fragmenting education, care, research, regulation, and reimbursement. This editorial argues for the abolition of that divide and the creation of a unified discipline of brain medicine. Drawing on advances in systems neuroscience, artificial intelligence, and neuromodulation, I present a comprehensive critique of the dual-specialty model and propose an integrated alternative. The editorial synthesizes data from brain network research, the lived inefficiencies of medical training and healthcare delivery, and the constraints placed on innovation by legacy frameworks. I highlight how computational psychiatry, neurobiology, and regulatory reform can converge to enable a model that is mechanistically grounded and clinically effective. Through two conceptual tables, I demonstrate the shared circuitry across traditionally labeled psychiatric and neurological conditions, and the system-wide efficiencies gained through integration. The future of brain care lies not in silos, but in circuits, and it is time our institutions, educators, payers, and innovators catch up with science. This is a call to unify how we understand, teach, regulate, and treat the brain not only for administrative elegance but also for clinical reality, therapeutic progress, and human dignity.

## Editorial

The false divide

The mind and brain are not separate, nor should the medical disciplines that attempt to understand and heal them be. Yet modern medicine remains shackled by a 19th-century divide: neurology on one side, psychiatry on the other. This division, entrenched in culture, training, reimbursement, diagnostics, and therapeutics, has no basis in neuroscience and no justification in clinical reality. It is time to abolish both psychiatry and neurology as separate entities and to unify them into a single field: brain medicine.

As a neurologist, neuropsychiatrist, neuroscientist, therapeutics developer, and educator at all levels of medicine, I have lived and witnessed the absurdity and harm of this bifurcation. The consequences ripple from medical school to bedside, from bench to policy. No other organ system is divided based on interpretive frameworks rather than biological and physiological mechanisms. In cardiology, we have electrophysiologists and heart failure specialists - different lenses on the same organ system but not ontologically separate disciplines. We do not send arrhythmias to one field and chest pain to another based on their emotional or mechanical components. Critically, the same organ system is the focus, and the interpretive framework, be it electrical or hemodynamic, does not necessitate ontologically separate disciplines. Endocrinologists manage diabetes whether it presents with mood changes or peripheral neuropathy. In all these specialties, the organ system comes first, and complexity is embraced rather than carved into artificial categories. Yet, we treat mood and cognition as if they are not functions of the brain. The notion that there are "mental" and "neurological" disorders is a linguistic relic, one that misguides diagnosis, delays care, and inhibits innovation.

In this editorial, I argue that the longstanding division between psychiatry and neurology is scientifically indefensible and clinically harmful. I present a comprehensive case for replacing these siloed specialties with a unified discipline of brain medicine, one that is rooted in systems neuroscience, structured for educational and regulatory efficiency, and aligned with real-world care and innovation.

Neuroscience and machine learning: collapsing legacy boundaries

Modern neuroscience and artificial intelligence are converging on a singular truth: the brain is an adaptive information-processing system whose dysfunction manifests across traditionally psychiatric and neurological lines. The longstanding division between these disciplines is not supported by systems biology, computational modeling, or clinical observation. It is a cultural relic, one increasingly contradicted by science.

The rise of machine learning and artificial neural networks has not only revolutionized technology but also illuminated the inner workings of the human brain. Like advanced computing systems, the brain processes inputs, applies learned rules, generates predictions, and adapts through feedback. This is not just metaphorical; it reflects shared principles of signal propagation, reinforcement learning, and error correction that underlie both artificial and biological systems.

Through computational psychiatry and systems neuroscience, we are beginning to map brain dysfunction with mathematical precision [1]. These fields reveal that abnormalities in prediction error, salience attribution, cognitive control, and affective bias are not confined to one side of the psychiatry-neurology divide. They are features of disrupted information processing, observed across depression, epilepsy, Parkinson’s disease, schizophrenia, and anxiety alike. If depression and epilepsy can both be modeled as failures in signal timing or feedback regulation, what sense does it make to treat them within separate professional silos?

Network neuroscience further erodes these boundaries. The default mode network, central executive network, and salience network underlie both cognitive and affective regulation (Table 1) [2]. Disorders of these systems manifest as Alzheimer’s, post-traumatic stress disorder, autism, or frontotemporal dementia, diagnoses arbitrarily labeled “neurologic” or “psychiatric.” These are not diseases of mind or body. They are diseases of circuits, systems, and signaling.

**Table 1:** Shared brain networks across psychiatric and neurological conditions This author-created table illustrates how major intrinsic brain networks contribute to both neurological and psychiatric conditions, challenging the traditional separation between the two fields. Each row presents a core brain network implicated in both domains, alongside specific disorders historically categorized as either neurological or psychiatric. The final column provides an integrative interpretation based on systems neuroscience, revealing how dysfunction within each network can produce diverse but mechanistically related clinical manifestations. This synthesis underscores the need for a unified brain medicine approach grounded in shared circuitry rather than legacy classifications. ADHD, attention-deficit/hyperactivity disorder; ASD, autism spectrum disorder; BPD, borderline personality disorder; FND, functional neurological disorder; OCD, obsessive-compulsive disorder; PD, Parkinson’s disease; PTSD, post-traumatic stress disorder; TLE, temporal lobe epilepsy

Brain Network	Traditionally Neurological Disorders	Traditionally Psychiatric Disorders	Unified Interpretation
Default mode network	Alzheimer’s disease, epilepsy, migraine	Major depression, schizophrenia, PTSD	Disruption of self-referential processing and autobiographical memory
Salience network	Frontotemporal dementia, stroke	Schizophrenia, anxiety disorders, OCD	Aberrant threat detection, interoception, and salience attribution
Central executive network	Multiple sclerosis, Parkinson’s disease	ADHD, bipolar disorder, borderline personality disorder	Impaired working memory, decision-making, and attention regulation
Sensorimotor network	Tourette’s syndrome, motor epilepsy	Functional movement disorder, somatic symptom disorder	Dysregulated sensorimotor integration and voluntary control
Limbic network	Temporal lobe epilepsy, autonomic disorders	Bipolar disorder, major depression, PTSD	Affective instability and emotional memory dysregulation
Cerebellar network	Ataxias, essential tremor	Autism spectrum disorder, schizophrenia	Dysmetria of thought, timing, and coordination of cognition
Thalamocortical network	Absence seizures, sensory integration disorders	Schizophrenia, attention disorders	Abnormal sensory filtering and consciousness regulation

Even symptomatology resists separation. Hallucinations appear in both Parkinson’s disease and schizophrenia. Executive dysfunction plagues both ADHD and early dementia. Cortical spreading depression informs both migraine and mood instability. Functional neurological disorders, once dismissed as “hysterical,” are now understood as disorders of network misfiring, not willpower [3].

Together, modern neuroscience and artificial intelligence converge on a shared conclusion: the brain is one system. Its disorders may manifest differently across individuals, but their roots lie in shared computational and physiological dysfunctions. A unified model of brain medicine must replace the fractured legacy of psychiatry and neurology.

Education: fragmentation from the start

The artificial divide begins early in medical training [4]. Undergraduate medical education carves the brain into "mental health" and "neuroanatomy" modules, reinforcing a dualistic model before students even touch a patient. Psychiatry clerkships rarely emphasize physical examination skills, neuroimaging interpretation, or biologically grounded models of dysfunction. Neurology rotations often neglect psychosocial, behavioral, and cultural contributors to disease. These tracks produce clinicians who are functionally and philosophically siloed, ill-equipped to address the spectrum of human brain dysfunction.

Graduate medical education amplifies the problem [4-5]. Residency programs offer virtually no required cross-training in neuropsychiatric integration. Psychiatry trainees may complete four years without managing a stroke or seizure, while neurology trainees may graduate without ever diagnosing schizophrenia or bipolar disorder. Fellowships are equally fragmented, entrenching niche subspecializations that deepen division rather than integration. Even combined programs in neuropsychiatry or behavioral neurology are rare, underfunded, and often treated as optional rather than essential.

As a former department chair and medical school dean of curriculum, I have seen the bureaucratic absurdities firsthand. Faculty appointments, curriculum committees, and departmental budgets reinforce boundaries that no longer serve educational or clinical goals. Medical students must navigate two languages, two cultures, and two ontologies, all for one brain. The American Board of Psychiatry and Neurology, despite its nominal unification, still supports divergent pathways, perpetuating this fragmentation in board certification, assessment, and continuing education.

Continuing medical education (CME) remains similarly divided, limiting the clinician’s ability to gain integrated competencies without pursuing parallel, duplicative tracks. In clinical practice, neurologists managing cognitive impairment are often underprepared for comorbid depression or anxiety. Psychiatrists treating psychosis may overlook seizure disorders, medication-induced cognitive decline, or neurodegenerative contributions. This divide compromises diagnostic accuracy, impairs treatment coordination, and perpetuates stigma.

At the health system level, this fragmentation breeds redundancy. Administrators must duplicate hiring, credentialing, and infrastructure across psychiatry and neurology, often to deliver overlapping services. Separate service lines may manage the same patient sequentially with conflicting approaches, generating inefficiency and confusion. A unified brain medicine education track could resolve these inefficiencies by preparing clinicians to practice at the intersections of cognition, emotion, and neurobiology, where most real-world patients reside.

Research and regulation: parallel silos, slowed progress

The implications extend to therapeutic development. Pharmacological innovation for brain disorders remains stagnant, in part, because studies are designed within silos. Trials for depression ignore comorbid epilepsy. Alzheimer's studies exclude patients with anxiety or psychosis [6]. Migraine research overlooks affective symptoms unless classified as "comorbid" [7]. This fragmentation fails to reflect real-world patients and limits translational impact [8].

Regulatory frameworks compound the issue. FDA drug review is split between psychiatric and neurological divisions [9] despite reviewing compounds that target shared pathways such as serotonin, glutamate, or GABA. This dichotomy not only slows down regulatory timelines but also introduces inefficiencies in guidance interpretation, complicates indication labeling, and impairs lifecycle planning. Sponsors are often forced to fragment their development programs across separate therapeutic areas, resulting in duplicated studies, inconsistent endpoints, and divergent filing strategies. These redundancies increase cost and time and, ultimately, delay access to promising therapeutics for patients who could benefit.

The burden is particularly acute in domains such as mood-cognition overlap, where a single therapy may affect multiple domains but must undergo a piecemeal review. Consolidating FDA’s neurology and psychiatry review divisions would allow for unified guidance, consistent standards, and cross-domain expertise that reflects how these therapies actually work. Integration would not only accelerate approvals but also reduce the burden on biopharma sponsors, streamline evidence generation, and promote broader, faster access to innovative interventions that transcend traditional diagnostic silos.

Practice and payers: inefficiencies and missed opportunities

In my work developing prescription digital therapeutics, I have seen firsthand how these tools defy conventional categorization [10]. A single digital intervention can target cognitive control, mood regulation, and sensory processing, domains straddling psychiatry and neurology. Yet reimbursement systems, regulatory pathways, and clinical guidelines often force artificial constraints. Clinicians must code visits according to outdated classification systems such as the Diagnostic and Statistical Manual of Mental Disorders (DSM) and International Classification of Diseases (ICD), which map poorly onto circuit-level dysfunctions and delay integration of personalized medicine.

Payers contribute to this fragmentation by imposing inconsistent coverage rules for therapies that do not fit neatly into one category. Neurostimulation, digital therapeutics, and cognitive-behavioral interventions all face reimbursement hurdles depending on whether they are labeled psychiatric or neurological, even when their mechanisms and indications overlap. These policies create structural disincentives for the development and deployment of integrated care models. Payers may require separate documentation, coding, and justification for a therapy’s neurological and psychiatric benefits, adding administrative burden, fragmenting care delivery, and limiting access for patients with comorbid conditions.

The impact on innovation is equally profound. Developers must navigate disconnected formularies, duplicative review panels, and fragmented benefit management systems. As a result, promising brain health solutions may fail to achieve meaningful market penetration, not due to lack of efficacy but because of an outdated reimbursement architecture. Conversely, a unified brain medicine framework would enable value-based coverage for transdiagnostic interventions, incentivize integrative outcomes, and encourage earlier, broader adoption of tools that promote brain resilience and functional recovery.

The path forward must include not only clinical integration but also financial and operational alignment across specialties. Payers have the opportunity to lead this transformation by unifying medical necessity criteria, aligning quality metrics, and supporting bundled payments for brain health interventions that span cognitive, affective, and behavioral domains. True reform will require evidence-based models that capture whole-person brain outcomes, replacing fragmented codes with unified metrics of impact.

Entrepreneurialism and innovation: breaking through the divide

Entrepreneurs at the intersection of technology and neuroscience increasingly recognize the futility of the psychiatry-neurology divide. Startups developing AI diagnostics, wearable neurotrackers, or cognitive-behavioral digital interventions routinely run into outdated frameworks that force them to define their solution as either "mental health" or "neurological," when in reality it is neither and both. The market is hungry for solutions that reflect how the brain actually works, not how medicine has chosen to categorize its dysfunctions.

This structural misalignment stifles innovation at every level. Companies must choose between FDA divisions, reimbursement codes, and clinical trial endpoints that often poorly match the intended function of their tools. A neurotechnology device that improves attention, memory, and mood must be wedged into a narrow “indication” lane for regulatory review, even though its mechanism spans multiple domains. Similarly, venture-backed platforms offering cognitive, neurobehavioral therapeutics for chronic pain or migraine face inconsistent payer coverage depending on whether their benefits are interpreted as psychiatric or neurologic.

Venture capital is beginning to respond, favoring transdiagnostic solutions and mechanistic platforms over traditional “indications.” Investors increasingly value neuroscience-informed strategies that target brain circuits and cognitive-affective processes rather than chasing single-entity approvals. However, entrepreneurs are still constrained by legacy structures that make commercialization and scaling of truly integrative tools risky and uncertain. Until academic medicine, regulatory authorities, and payors catch up, innovation will remain outpaced by bureaucracy.

A new framework, brain medicine, can provide the legitimacy and infrastructure for integrated innovation. By aligning scientific insight with clinical pathways and payer logic, it offers a home for interventions that target brain function holistically. It also signals to startups and their funders that the future of neurotechnology and digital health is not defined by categories of the past but by mechanisms, outcomes, and impact across the full spectrum of brain health.

A vision for unified brain medicine

Unifying psychiatry and neurology into brain medicine would yield immediate and long-term efficiencies. Training pipelines could be streamlined, with integrated rotations in neurobehavioral health and shared foundational knowledge in circuitry, development, and systems neuroscience. Redundant medical school curriculum, residency structures, board certifications, and CME credits could be consolidated into a single, coherent framework focused on brain systems. Professional societies such as the American Psychiatric Association (APA) and the American Academy of Neurology (AAN) could merge or, at minimum, coordinate joint initiatives, policy advocacy, and annual scientific meetings, pooling intellectual capital and financial resources to address complex brain health challenges.

A unified discipline of brain medicine would not merely be a merger. It would be a transformation, one that redefines how we understand, measure, and intervene in brain function. It would move away from symptom-based classification systems that fail to reflect neurobiological mechanisms toward a model rooted in neural circuitry, developmental trajectories, and individual variability. It would embrace biological psychiatry not as a niche but as a foundation upon which to build deeper integration with digital phenotyping, psychoneuroimmunology, computational modeling, and real-time data capture.

Brain medicine would also embody preventive and promotive health, not just disease management. It would enable clinicians to identify early warning signs of brain dysfunction across cognitive, affective, and behavioral dimensions, regardless of legacy diagnostic labels. A brain medicine model would allow interventions to be personalized not only by symptoms but also by biomarkers, environmental context, and adaptive capacity. It would create space for hybrid therapeutics that include software, behavior, pharmacology, and lifestyle, and evaluate them on outcomes that matter most: functioning, autonomy, and resilience.

Crucially, this transformation would also signal to health systems, regulators, and innovators that we are ready to practice what neuroscience has long revealed - that the brain is not two domains, but it is one dynamic, integrated organ that governs all thoughts, emotions, movements, and identities. A brain medicine framework does not eliminate specialization. Rather, it recalibrates it toward shared mechanisms, team-based care, and a common purpose - to preserve and restore brain health across the lifespan (Table 2).

**Table 2:** Systemic efficiencies gained from integrating psychiatry and neurology into a unified brain medicine framework This author-created table summarizes the inefficiencies currently observed across key domains of the healthcare ecosystem due to the historical and artificial division between psychiatry and neurology. Each domain - spanning education, research, regulation, payer policy, clinical practice, entrepreneurial innovation, and societal perception - experiences fragmentation that hinders effective care, slows innovation, and increases costs. The right-hand column describes the projected efficiencies that would emerge from adopting a unified discipline of brain medicine. These improvements include curricular integration, transdiagnostic funding models, consolidated regulatory review, unified reimbursement mechanisms, streamlined clinical pathways, and enhanced public understanding. The proposed transformation aligns modern neuroscience with the realities of healthcare delivery, promoting a coherent, systems-based approach to brain health. FDA, U.S. Food and Drug Administration; NIH, National Institutes of Health

Domain	Current Fragmentation	Unified Brain Medicine Efficiency
Medical education	Separate rotations, curricula, examinations, and board certifications for psychiatry and neurology.	Integrated curricula, consolidated board examinations, and cross-disciplinary clinical training.
Research	Siloed NIH funding streams, few cross-specialty grants, and discipline-specific research agendas	Transdiagnostic, circuit-based funding models that reflect modern neuroscience
Regulation	Split FDA review divisions for psychiatric and neurological therapies with duplicative processes	Consolidated regulatory pathways for brain-active therapeutics across the spectrum
Payer coverage	Reimbursement varies by labeling, with mental health and neurological treatments funded inconsistently	Unified, value-based coverage for all brain-system interventions, digital and pharmacological
Clinical practice	Separate service lines, specialist referrals for overlapping conditions, and fragmented patient experience	Streamlined care with holistic brain health providers managing across domains
Entrepreneurialism	Categorization bottlenecks hinder development and reimbursement of neurotech and digital tools	Clearer innovation pathways and regulatory recognition for integrative solutions
Society	Persistent stigma and confusion due to artificial distinctions between mental and neurological disorders	Reduced stigma and increased public understanding of brain health as a unified concept

A call to action

To achieve this, we must begin with education. Medical schools should replace outdated silos with integrated brain health curricula that bridge neuroscience, behavior, and clinical application. Students should learn the structure and function of brain networks before memorizing DSM or ICD categories, and they should practice thinking in terms of systems, mechanisms, and dynamic regulation. Residency programs should evolve into unified tracks in brain medicine, with rotations across neurology, psychiatry, geriatrics, neurodevelopment, and integrative modalities including digital therapeutics, neuromodulation, and neurotechnology. Continuing education must prioritize circuit-level knowledge, symptom-network mapping, and emerging therapeutic platforms.

Academic centers should follow suit, rethinking their organizational charts and administrative boundaries. Merged departments of brain medicine can create interdisciplinary labs, clinical centers, and innovation hubs. Research institutes must break down barriers between mental health and neurology grants, allowing principal investigators to pursue transdiagnostic hypotheses and integrated biomarkers. Journals must welcome cross-domain submissions and editorial leadership that recognizes the need for a new scientific lexicon.

Policy-makers and payers must also evolve. They should support unified diagnostic and billing codes that reflect circuit dysfunction and functional outcomes. Bundled payments and brain health pathways should reimburse for interventions based on efficacy and value, not their categorization. Regulators must issue cross-division guidance for transdiagnostic therapeutics, especially as combination interventions emerge that span mood, cognition, and behavior. Industry sponsors must be encouraged to design studies that reflect real-world complexity, not artificially pure diagnostic cohorts, and to seek labels that reflect mechanisms and populations, not only legacy indications.

This transformation will require bold leadership and institutional humility. However, the cost of inaction is immense. Patients with overlapping psychiatric and neurological symptoms will continue to fall through the cracks; misdiagnoses will persist; effective treatments will be delayed or denied; clinicians will be forced to work in fragmented systems, undermining their training, compassion, and innovation; and the lifeblood of medical progress will be stifled by bureaucracy, confusion, and risk aversion.

In conclusion, the brain is one, and hence our approach to it must also be one. It is time to abolish psychiatry and neurology as separate entities and replace them with a unified discipline of brain medicine. This is not about erasing specialties but about healing the fracture between them. It is about aligning our science, our training, our care delivery, and our innovation pipeline with the reality of the human brain, as the future of medicine depends on it.
